# Nuclear and Mitochondrial Circulating Cell-Free DNA Is Increased in Patients With Inflammatory Bowel Disease in Clinical Remission

**DOI:** 10.3389/fmed.2020.593316

**Published:** 2020-12-14

**Authors:** Zuzana Vrablicova, Kristina Tomova, Lubomira Tothova, Janka Babickova, Barbora Gromova, Barbora Konecna, Robert Liptak, Tibor Hlavaty, Roman Gardlik

**Affiliations:** ^1^5th Department of Internal Medicine, Sub-department of Gastroenterology and Hepatology, Faculty of Medicine, Comenius University, University Hospital Bratislava, Bratislava, Slovakia; ^2^2nd Department of Gynecology and Obstetrics, Hospital of F. D. Roosevelt, Slovak Medical University, Banska Bystrica, Slovakia; ^3^Faculty of Medicine, Institute of Molecular Biomedicine, Comenius University, Bratislava, Slovakia; ^4^Department of Clinical Medicine, University of Bergen, Bergen, Norway; ^5^Faculty of Medicine, Institute of Physiology, Comenius University, Bratislava, Slovakia

**Keywords:** extracellular DNA, deoxyribonuclease (DNase), remission, Crohn's disease, ulcerative colitis

## Abstract

**Background:** The role of cell-free DNA (cfDNA) in the pathogenesis of inflammatory bowel disease (IBD) has been recently suggested. The aim of this study was to analyze circulating cfDNA and deoxyribonuclease (DNase) activity in IBD patients in clinical remission.

**Materials and Methods:** Plasma and serum were obtained from 72 patients with Crohn's disease and 28 patients with ulcerative colitis. Total cfDNA, nuclear DNA (ncDNA), mitochondrial DNA (mtDNA) and DNase activity were measured.

**Results:** IBD patients showed higher levels of both ncDNA and mtDNA compared to healthy controls. Concentration of ncDNA was higher in males compared to females, including patients and healthy controls. However, unlike males higher amount of ncDNA was found in female IBD patients compared to healthy controls. DNase activity was significantly lower in male IBD patients compared with healthy controls. In addition, there was a negative correlation between DNase activity and ncDNA levels in male IBD patients.

**Conclusions:** Herein we present increased amount of circulating ncDNA and mtDNA in IBD patients in clinical remission. Thus, unlike total cfDNA, circulating ncDNA and mtDNA might not represent the optimal biomarkers of disease activity. This is also the first report on sex difference in circulating ncDNA levels, possibly associated with lower DNase activity in males.

## Introduction

Inflammatory bowel disease (IBD) is a group of multifactorial, autoimmune disorders with multifactorial etiology, which includes Crohn's disease (CD) and ulcerative colitis (UC). Despite extensive research, the detailed pathogenesis has not been sufficiently explained yet. Current therapy is mostly based on ameliorating inflammatory processes, which does not cover the whole complexity of the disease and inevitably leaves a large group of patients without efficient therapy. Apart from microbiome-directed studies, novel approaches to uncover the pathogenesis of IBD include the analysis of cell-free (or extracellular) nucleic acids.

Increased concentrations of circulating cell-free DNA (cfDNA) has been previously shown to be associated with multiple inflammatory disorder, including sepsis or hepatorenal injury (1, 2). A number of studies have suggested a role of cfDNA in IBD. The role of cell-free nucleic acids and their clinical relevance in IBD has been recently reviewed by Kubiritova et al. (3). To this date, a number of animal studies have proved the role of cfDNA in IBD. CfDNA is able to bind to toll-like receptor 9 (TLR9) and activate the pathway leading to inflammatory responses. This designates cfDNA a potential marker of inflammation. However, several experiments on mice have paradoxically confirmed the protective role of these pathways in IBD (4, 5). On the other hand, activation of TLR9 significantly exacerbated mouse colitis in a different study (6). In addition, a positive correlation between plasma cfDNA concentration and severity of colitis has been shown (7). Interestingly, concentrations of circulating cfDNA negatively correlated with deoxyribonuclease (DNase) activity in the colon tissue (8).

The above mentioned findings, however, have been so far confirmed only in a few patient studies. Circulating total cfDNA concentrations in UC patients were significantly positively correlated with the clinical severity of UC (7). Increased levels of circulating cfDNA of mitochondrial origin (mtDNA) were observed in a variety of inflammatory diseases and acute injuries (9–11). However, there is only one study showing increased levels of circulating mtDNA in active IBD patients. MtDNA levels significantly correlated with disease markers and activity (12). In addition, a reduced serum DNase I activity was also observed in patients with IBD (13). Thus, the nature of circulating cfDNA in IBD patients has not been extensively studied yet. The aim of this study was to analyze the concentrations of circulating cfDNA and the activity of DNase in IBD patients in clinical remission and look for potential sex differences.

## Materials and Methods

### Patients and Controls

One hundred IBD patients were recruited from outpatient setting from the Gastrointestinal Unit of the 5th Department of Internal Medicine, University Hospital Bratislava between September 2016 to June 2017. Diagnosis of CD and UC was made upon endoscopic, histologic and clinical findings according to standard clinical, endoscopic and histological criteria. At the time of sampling, all CD patients had the Crohn's Disease Activity Index score equal or below 150 and Harvey-Bradshaw index score below 5. Patients with UC had the Partial Mayo Score below 2. All patients were on ongoing treatment that included one or more of the following drugs: infliximab, vedolizumab, ustekinumab, adalimumab, azathioprine, mesalazine. Five CD patients and 4 UC patients were current smokers. Twenty-nine healthy non-smokers with no history of IBD and no acute or chronic illness were recruited as non-IBD controls. Informed consent was obtained from all participants prior to their inclusion in the study. Characteristics of patients and healthy controls are listed in Table 1. The study has been approved by the local ethics committee and has been performed in accordance with the ethical standards laid down in the 1964 Declaration of Helsinki and its later amendments.

**Table 1:** Characteristics of the study groups.

	**Controls**	**CD**	**UC**
*n*	29	72	28
Age	27 (18–46)	41 (22–69)	40 (21–71)
Females %	59	39	50
BMI	22.6	24.3	24
CDAI	–	≤ 150	–
HBI	–	<5	–
pMayo	–	–	<2

*CD, Crohn's disease; UC, ulcerative colitis; BMI, body mass index; CDAI, Crohn's disease activity index; HBI, Harvey-Bradshaw index; pMayo, partial Mayo score*.

### Blood Samples Processing

Samples were taken from IBD patients during their outpatient visit. Healthy volunteers provided their blood samples at the Faculty of Medicine, Comenius University, Bratislava. Venous blood was taken into EDTA and serum Vacuette tubes (Greiner Bio-One, Frickenhausen, Germany) and processed within 2 h. Samples were centrifuged at 1,600 g for 10 min at 4°C, the supernatant was transferred into a new 15-mL tube and centrifuged again at 16,000 g for 10 min at 4°C. The resulting supernatant was used as cell-free plasma and serum, respectively.

### Extraction and Measurement of cfDNA

DNA was extracted from 200 μL cell-free plasma samples using QIAamp DNA Blood Mini Kit (Qiagen, Hilden, Germany) following the manufacturer's instructions. The concentration of total cfDNA was measured using Qubit Fluorometer and Qubit dsDNA HS Assay Kit (Invitrogen, Los Angeles, California, USA) and expressed as ng of DNA per mL of plasma.

### Quantitative PCR

Quantitative PCR was used for the quantification of ncDNA and mtDNA in cfDNA extracted from plasma of both sets. Quantitative PCR was performed on Mastercycler realplex 4 (Eppendorf, Hamburg, Germany) using the SsoAdvanced Universal SYBR Green Supermix (Bio-Rad, Hercules, California, USA) with the following program: 1 cycle of 2 min at 95°C, followed by 45 cycles of 95°C for 15 s for denaturation and 60°C for 1 min for annealing. After completion of all cycles a melting curve was obtained. Primers were designed for the amplification of part of human beta globin gene to quantify ncDNA (F: GCT TCT GAC ACA ACT GTG TTC ACT AGC, R: CAC CAA CTT CAT CCA CGT TCA CC) and part of human cytochrome c oxidase gene to quantify mtDNA (F: CAT AAA AAC CCA ATC CAC ATC A, R: GAG GGG TGG CTT TGG AGT). The amount of ncDNA and mtDNA was expressed as number of copies per mL of plasma.

### DNase Activity

DNase activity in plasma samples was measured by single radial enzyme diffusion method using 1% agarose gel (20 mM Tris–HCl, pH 7.5, 2 mM MgCl_2_, 2 mM CaCl_2_), which contained DNA isolated from chicken liver (7 ml of 5 mg/ml DNA in 100 ml of gel). From each serum sample 1 μl was pipetted into the gel and incubated over night at 37°C in the dark. Dilutions of DNase I (Qiagen, Hilden, Germany) of known concentrations were used for creating the calibration curve. The diameters of circles were measured by ImageJ software (Madison, Wisconsin, USA). DNase activity was expressed as Kunitz units (KU) per mL of plasma.

### Statistical Analysis

All data were analyzed and presented using GraphPad Prism 6.0 (GraphPad Software, La Jolla, California, USA) software using unpaired *t*-test and one-way ANOVA with Tukey's *post hoc* test, where appropriate. *P*-values of < 0.05 were considered significant. All data are presented as mean ± SD.

## Results

### Increased Circulating ncDNA and mtDNA in IBD Patients

**Figure 1:**
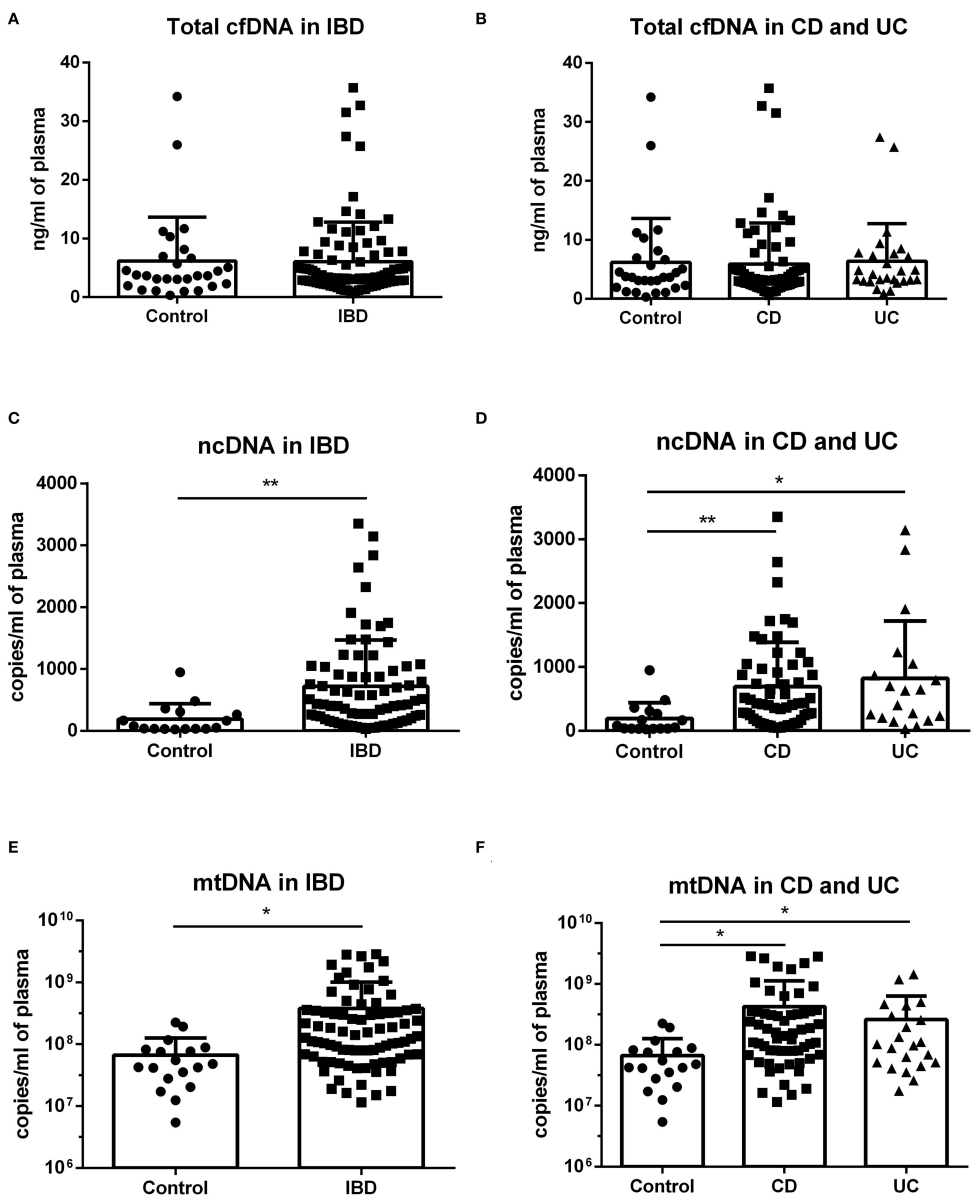
Concentration of total cfDNA, ncDNA and mtDNA in IBD patients and controls. No difference in total cfDNA between healthy controls and patients was found **(A,B)**. IBD patients showed higher levels of both ncDNA and mtDNA compared to healthy controls (**C**: *p* = 0.0061 and **E**: *p* = 0.0387). The difference was also seen in both subgroups of CD and UC patients (**D**: *p* = 0.0062 for CD and 0.0105 for UC against control and **F**: *p* = 0.0345 for CD and *p* = 0.0365 for UC against control). *P < 0.05, **P < 0.01.

When the subject groups were analyzed regardless of sex, the difference between healthy controls and patients was not present in total cfDNA (Figures 1A,B). However, IBD patients showed higher levels of both ncDNA and mtDNA compared to healthy controls (Figures 1C,E). This difference was seen in both CD and UC patients (Figures 1D,F).

### Sex Difference in Circulating ncDNA

**Figure 2:**
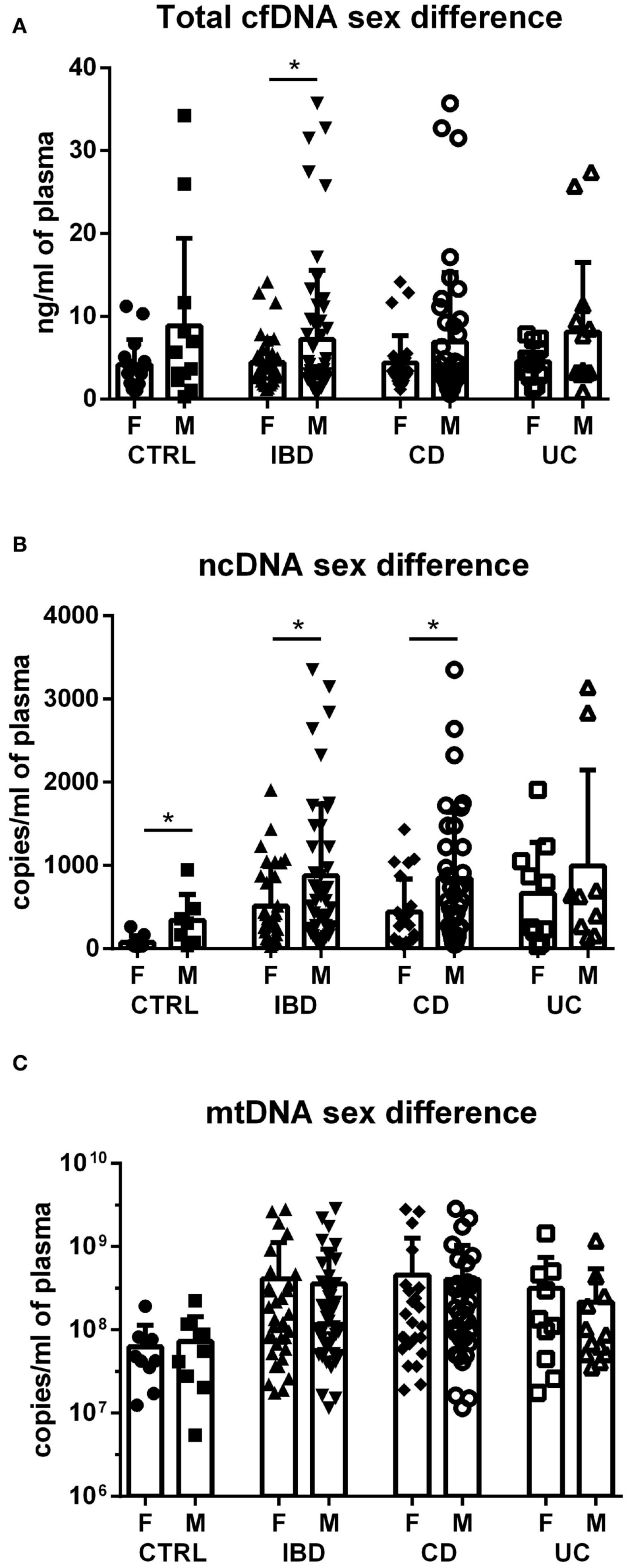
Sex differences in cfDNA, ncDNA and mtDNA. Higher total cfDNA was found in male IBD patients compared with female patients (*p* = 0.0478); however, this difference was not found in the subgroups of CD and UC **(A)**. Concentration of ncDNA is higher in males compared to females in healthy controls (*p* = 0.0274), IBD patients (*p* = 0.0345) and CD subgroup (*p* = 0.0309) **(B)**. No sex difference was found in mtDNA in any of the groups **(C)**. *P < 0.05.

Although no sex difference was found in total cfDNA and mtDNA levels in any of the groups (Figures 2A,C; except for cfDNA in all IBD), absolute concentration of ncDNA is significantly higher in males compared to females (Figure 2B). This is true for CD patients as well as IBD as a whole. In UC patients, the difference is not significant, likely due to lower number of subjects. This sex difference is also obvious in healthy controls with males showing higher levels of ncDNA.

### Increased Circulating ncDNA and mtDNA in Female IBD Patients

**Figure 3:**
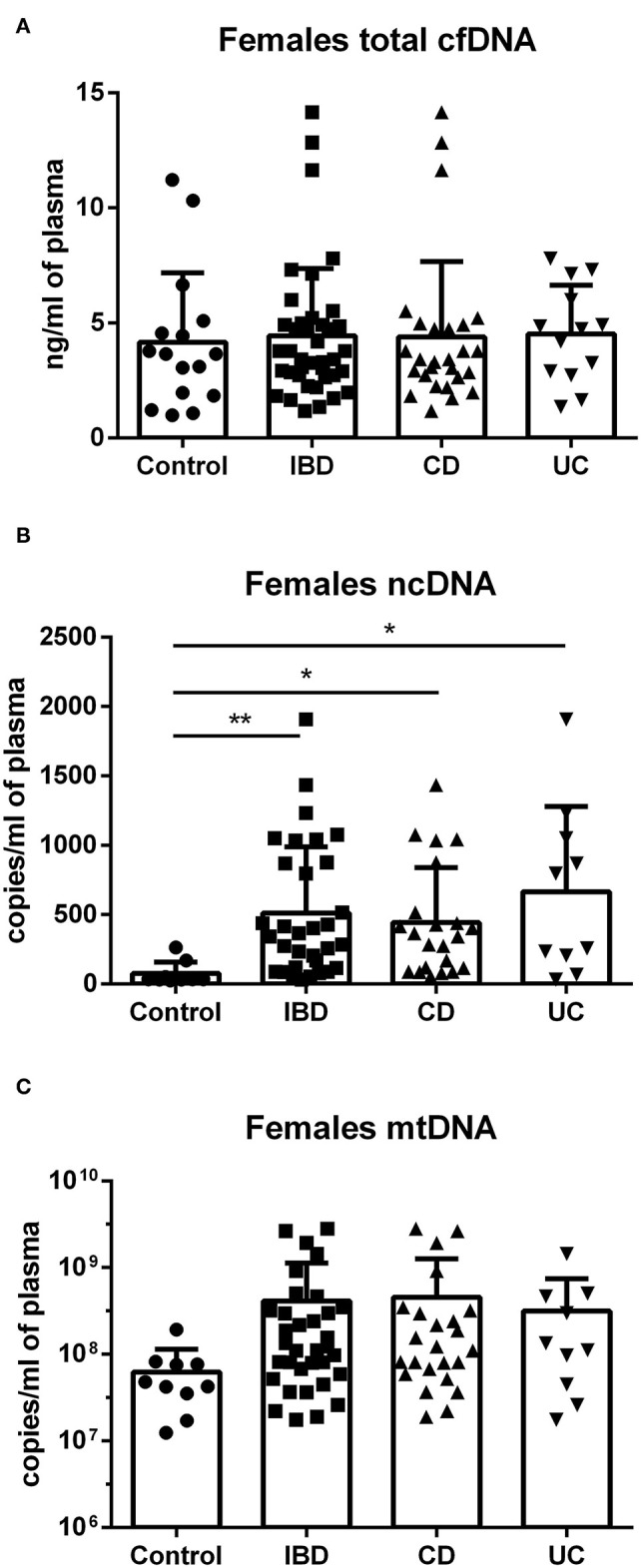
Total cfDNA, ncDNA and mtDNA in females. No difference was found between groups in total cfDNA **(A)**. Higher amount of ncDNA was found in female IBD patients (*p* = 0.0098), including both CD (*p* = 0.0108) and UC (*p* = 0.0110) compared to healthy controls **(B)**. Similar trend is seen in mtDNA, although the differences were not significant **(C)**. *P < 0.05, **P < 0.01.

**Figure 4:**
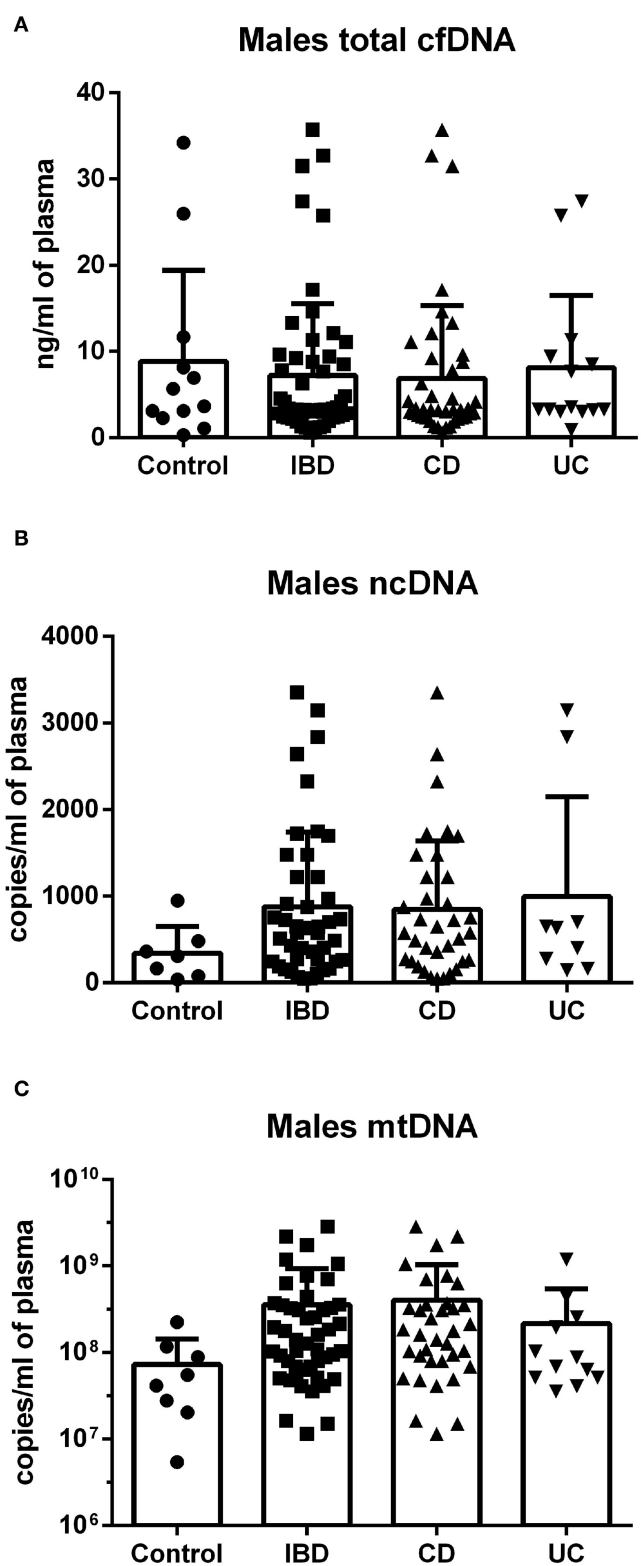
Total cfDNA, ncDNA and mtDNA in males. No significant differences between groups were shown in cfDNA **(A)**, although a clear trend toward higher ncDNA and mtDNA in IBD patients compared with healthy controls can be seen **(B,C)**.

In females, no difference was found between groups in total cfDNA (Figure 3A); on the other hand, a significantly higher amount of circulating ncDNA was found in female IBD patients (both CD and UC) compared to healthy controls (Figure 3B). Although not significant, this finding was seen in mtDNA as well (Figure 3C). In males, no significant differences between groups were shown, although the trend toward higher ncDNA and mtDNA in IBD patients compared with healthy controls can be seen (Figures 4A–C).

### Lower DNase Activity in Male IBD Patients

**Figure 5:**
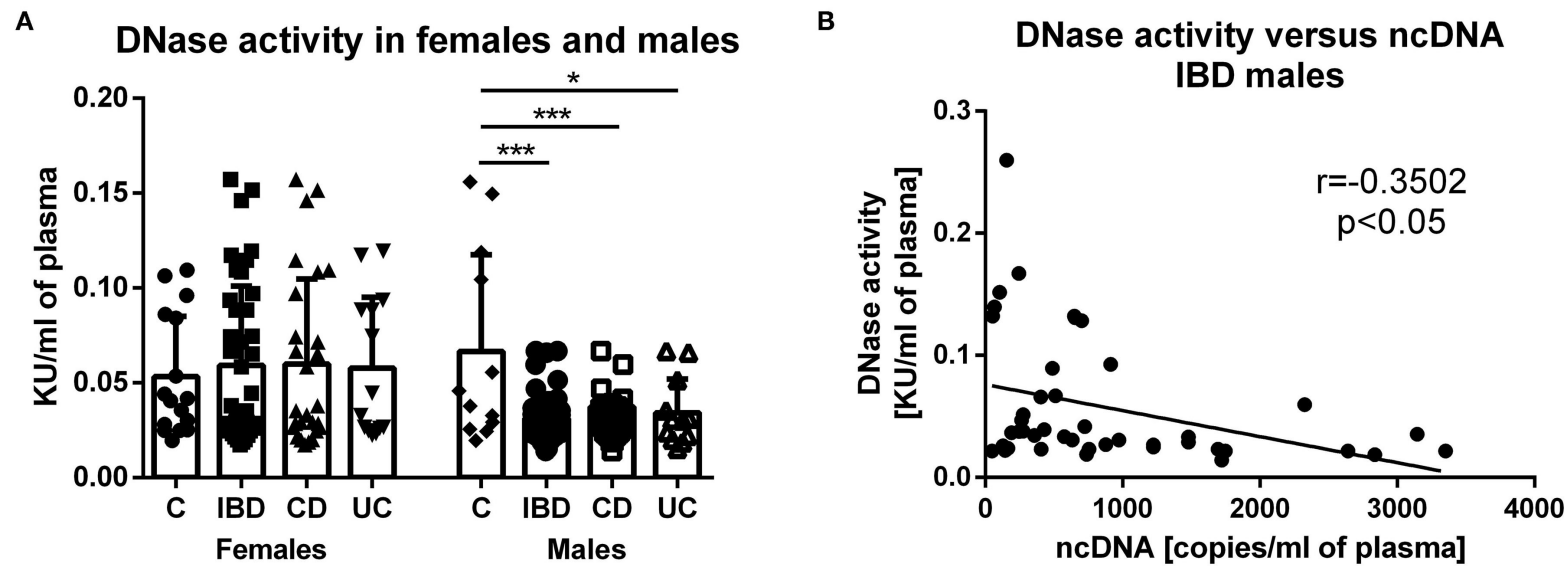
DNase activity in healthy controls and patients. In females, no difference was found between groups. DNase activity was lower in male IBD patients (*p* = 0.0001), including both CD (*p* = 0.0002) and UC (*p* = 0.0497) compared with healthy controls **(A)**. Negative correlation was found between DNase activity and concentration of ncDNA in male IBD patients (*p* = 0.0198) **(B)**. *P < 0.05, ***P < 0.001.

In females, no difference was found between groups in DNase activity (Figure 5A). Interestingly, DNase activity was significantly lower in male IBD patients (both CD and UC) compared with healthy controls. In addition, there is a significant negative correlation between DNase activity and ncDNA in male IBD patients (Figure 5B). However, no correlation was found between DNase activity and any form of DNA in female IBD patients.

## Discussion

Our study is the first one analyzing the amount of circulating total cfDNA, ncDNA and mtDNA in human IBD patients in clinical remission. It was previously found that total circulating cfDNA, as well as mtDNA is increased in active IBD patients (7, 12). This clearly shows the potential of these molecules to serve as biomarkers of disease severity and potentially as therapeutic targets.

It has been shown that circulating mtDNA can activate various inflammatory responses via TLR9 receptors, including NLRP3 inflammasomes and neutrophils (14, 15). Boyapati et al. proved that deletion of the *Tlr9* gene results in the attenuation of acute colitis in mice, which indicates that mtDNA-TLR9 signaling represents a targetable pathway in IBD (12). Herein we present that the amount of circulating ncDNA and mtDNA is also increased in non-active IBD patients in clinical remission. Thus, unlike total cfDNA, circulating ncDNA and mtDNA might not represent the best biomarkers of disease activity, since their amounts are higher even in IBD patients in clinical remission compared with healthy controls.

Although absolute concentration of ncDNA was higher in males than females, the difference between healthy controls and IBD patients is only significant in females. This is mainly because healthy male controls had values which are higher compared with healthy female controls and, in fact, are close to male IBD patients. Thus, circulating ncDNA amount clearly differentiates between non-IBD and IBD individuals, but only in females. This might be due to the fact that circulating levels of neutrophil extracellular traps (NETs) are higher in males compared with females (16). NETs represent one of the sources of ncDNA. NcDNA is a potentially pro-inflammatory molecule playing role in initiating and sustaining the innate immune response (17). This is the first study that shows the sex difference in circulating ncDNA levels in IBD patients. Our findings are in accordance with a study by Meddeb et al. that has shown higher ncDNA levels in males compared to females and no sex difference in mtDNA in healthy individuals (18).

One of the possible limitations of this study is the age difference between healthy control group and IBD patients (mean age: 27 for controls, 41 for CD, 40 for UC). Age was previously found to be a significant factor influencing the levels of ncDNA, but not mtDNA in healthy individuals (18). Nevertheless, no significant correlation was found between age and cfDNA, ncDNA or mtDNA concentrations in any of the subject populations in this study, even if sex was considered (data not shown). Another limitation of our current study is the fact that only IBD patients in clinical remission were enrolled. However, data from the literature provide some insight into the nature of cfDNA in active IBD patients. Our study focused only on patients in clinical remission. Future studies should include patients with various disease activity scores and long-term sampling of the same patients to monitor the dynamics of the cfDNA in active and non-active stages of the disease.

The group of Monteleone has shown that NETs, which include both ncDNA and mtDNA, increased in colon mucosa and sustain inflammatory processes in UC (17). On the other hand, circulating gut-derived cfDNA has not proved higher in IBD patients (19). The authors did not find intestinal cfDNA in plasma of healthy individuals. In addition, active IBD patients only had minimal amounts of circulating intestinal cfDNA, which were not significantly different from healthy controls. This finding indicates that circulating total cfDNA is likely not of intestinal origin, although additional studies are lacking. Our group has recently shown that total cfDNA increased with the stage of colitis and mtDNA and ncDNA was not significantly different compared to control mice (20).

It has been reported that DNase I deficiency promotes susceptibility to autoimmune disorders (21). In addition, a decreased DNase I activity has been previously observed in active IBD patients (13). Herein we present that this finding is also valid for IBD patients in clinical remission; however, this is only true for male patients. Sex difference in serum DNase activity of IBD patients has not been reported before. We also reported a significant negative correlation between DNase activity and circulating ncDNA in male IBD patients, suggesting a potential key role of this finding in the pathogenesis of male IBD. Injection of DNase was effective in experimental treatment of sepsis (1) and hepatorenal injury (2). However, no significant therapeutic effect of intravenous DNase I injection was proved in mouse colitis, possibly due to rapid half-life of the enzyme in the circulation (22). The current study also contributes to the knowledge on the mechanisms of DNase action from the view of sex differences.

## Data Availability Statement

The raw data supporting the conclusions of this article will be made available by the authors, without undue reservation.

## Ethics Statement

The studies involving human participants were reviewed and approved by Ethics committee of the University Hospital in Bratislava. Approval No. EK 104/2017. The patients/participants provided their written informed consent to participate in this study.

## Author Contributions

Patient examination, sample collection and study supervision were performed by ZV and TH. Sample preparation and processing were performed by KT, BG, and RL. Data collection and analysis were performed by LT, BK, and JB. The manuscript was written and the funding acquired by RG. All authors contributed to the study conception and design and read and approved the final manuscript.

## Conflict of Interest

The authors declare that the research was conducted in the absence of any commercial or financial relationships that could be construed as a potential conflict of interest.
