# Supplementary figures and images for: Nuclear and Mitochondrial Circulating Cell-Free DNA Is Increased in Patients With Inflammatory Bowel Disease in Clinical Remission
Source: Front Med (Lausanne). 2020 Dec 14;7:593316. doi: 10.3389/fmed.2020.593316 (PMC7768081; doi:10.3389/fmed.2020.593316)

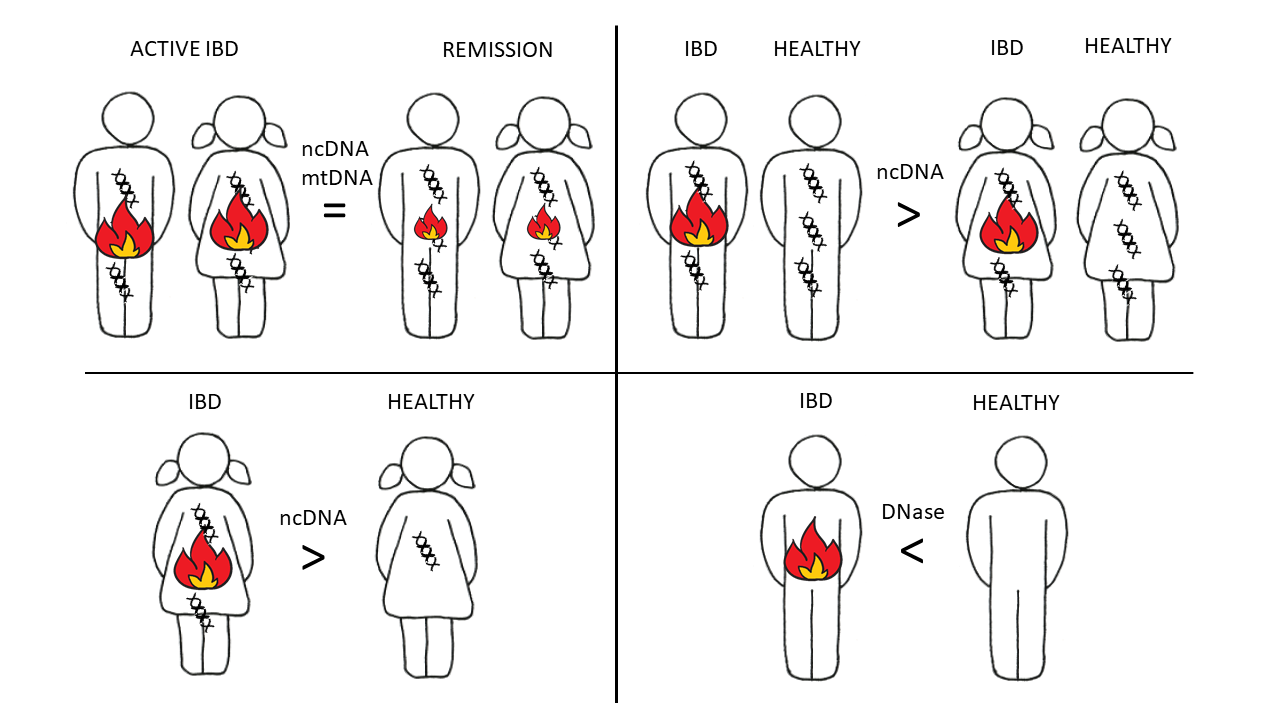

Supplement: Supplementary file 1 [file Image_1.TIF]
